# Comparison of the gene expression profile of testicular tissue before and after sexual maturity in Qianbei Ma goats

**DOI:** 10.1186/s12917-024-03932-0

**Published:** 2024-03-08

**Authors:** Jiajing Chen, Xiang Chen, Wei Guo, Wen Tang, Yuan Zhang, Xingzhou Tian, Yue Zou

**Affiliations:** 1 Key Laboratory of Animal Genetics, Breeding and Reproduction in the Plateau Mountainous Region, Ministry of Education, Guizhou University, Guiyang, 550025 China; 2 Guizhou Provincial Key Laboratory of Animal Genetics, Breeding and Reproduction, Guizhou University, Guiyang, 550025 China

**Keywords:** Qianbei Ma Goats, Testis, RNA-seq, DEGs, Male reproduction

## Abstract

**Background:**

With long-term research on the reproductive ability of Qianbei Ma goat, we found that the puberty of the male goats comes at the age of 3 months and reaches sexual maturity at 4 months,the male goats are identified as physically mature at 9 months and able to mate. Compared with other kinds of breeds of goats, Qianbei Ma goat is featured with more faster growth and earlier sexual maturity.Therefore, in order to explore the laws of growth of Qianbei Ma goat before sexual maturity(3-month-old)and after sexual maturity (9-month-old). The testicular tissue was collected to explore their changes in morphology through HE staining, the serum was collected to detect the hormone content, and the mRNA expression profile of the testis was analyzed by transcriptomics. In this way, the effect of testicular development on the reproduction of Qianbei ma goats was further analyzed.

**Results:**

The results showed that the area and diameter of spermatogenic tubules were larger at 9 months than 3 months, and the number of spermatocytes, interstitial cells, spermatogonia and secondary spermatocytes in the lumen of the tubules showed a similar trend. The appearance of spermatozoa at age 3 months indicated that puberty had begun in Qianbei Ma goats. The Elasa test for testosterone, luteinizing hormone, follicle stimulating hormone and anti-Müllerian hormone showed that the levels of these hormones in the serum at age 9 months were all highly significantly different than those at age 3 months (*P* < 0.01). There were 490 differentially expressed genes (DEGs) between the (|log2(fold change)| > 1 and p value < 0.05) 3-month-old and 9-month-old groups, of which 233 genes were upregulated and 257 genes were downregulated (3 months of age was used as the control group and 9 months of age was used as the experimental group). According to the GO and KEGG enrichment analyses of DEGs, *PRSS58*, *ECM1*, *WFDC8* and *LHCGR* are involved in testicular development and androgen secretion, which contribute to the sexual maturation of Qianbei Ma goats.

**Conclusions:**

Potential biomarker genes and relevant pathways involved in the regulation of testicular development and spermatogenesis in Qianbei Ma goats were identified, providing a theoretical basis and data support for later studies on the influence of testicular development and spermatogenesis before and after sexual maturity in Qianbei Ma goats.

## Background

As an important reproductive organ in male mammals, the testis plays a crucial role in the reproductive process of domestic animals. Testicular development varies in different periods, with large testicular development having a significant effect on spermatogenesis [1]. Testicular size was found to be significantly positively correlated with ejaculate volume, sperm concentration and sperm viability and negatively correlated with the percentage of abnormal spermatozoa in bovine, caprine, and porcine animals [2–5]. In addition, testicular size not only affects male reproductive performance but also affects litter size and litters per year [6]. Therefore, the development of testes is one of the most effective methods to evaluate the reproductive performance of a sire. Zhang found that the testicular tissue section of 30-day-old Changbai breeder boars had an obvious cord-like structure, only support cells and spermatogonia were observed in the varicocele, the lumen was not formed, and there was no spermatogenesis. The 210-day-old Changbai breeder boars had a substantial increase in the volume of the testis, and the diameter of the ductal lumen was obviously larger, with many round and elongated spermatogonia appearing in the official lumen; at the same time, the number of spermatogonia in the lumen was increased [7]. Studies have shown that after sexual maturity, small-tail Han sheep have the highest ejaculate volume, high semen density, high sperm survival rate, long sperm survival time outside, and good semen quality [8]. Testicular size is positively correlated with scrotal circumference. Yadav et al. ( 2019 ) found that the scrotal circumference of buffalo was significantly positively correlated with ejaculation volume, sperm motility and motility. The larger the scrotal circumference is, the better the semen quality is [9]. In addition, the testis is the site of synthesis of the androgenic steroid testosterone, which is essential for males to maintain secondary sexual characteristics and the skeletal muscular system and initiate spermatogenesis [10]. The major role of testosterone in spermatogenesis promotes the reorganization of the structure of the blood‒testis barrier to maintain its stability and induces the movement of spermatogonial cells of the thin-lineage stage through the blood‒testis barrier to the lumen of the seminiferous tubules [11]. Testosterone can alter the expression and post-translational modification of nearly 25 proteins, including DNA repair and RNA splicing, to maintain support cell spermatocyte adhesion and enhance the associated adhesion proteins between support cells and immature germ cells, preventing premature separation of round spermatocytes from support cells [12, 13]. The amount of testosterone secreted by male animals varies at different times during growth and development. Previous studies have demonstrated that testicular development has a major influence on the production and secretion of testosterone, which gradually increases from low to the highest level before and after sexual maturity [14]. Wei found that with age in male Liaoning cashmere goat kids the secretion level of testosterone in peripheral serum also increased gradually, and the secretion level after 30 days of age was divided into 2.23, 5, 7.94, 10.41, 13.45, and 16.16 times that of the neonatal level [15]. Testicular development has a strong influence on the production activities of subsequent male progenies; therefore, it is very important to investigate the impact of testicular development on male reproductive development.

The Qianbei Ma goat is one of the three excellent local goat breeds in Guizhou and displays roughage tolerance, strong disease resistance, good grouping, ease of keeping in captivity, a docile temperament, and strong adaptability [16],It has greater development potential and value.Based on the importance of testes for Qianbei Ma goat reproduction,In this experiment, we analyzed the mRNA expression profiles of the testes of Guizhou Qianbei Ma goats at 3 months of age (before sexual maturity) and 9 months of age (after sexual maturity) by transcriptomics, screened the differentially expressed genes in different periods, and explored the functions of the differentially expressed genes to provide basic data for the enhancement of the reproductive ability of Guizhou Qianbei Ma goats.

## Materials and methods

### Animal ethics

All animal experiments were carried out in strict accordance with the instructions provided by the Animal Care and Use Committee of the Laboratory Animal Ethics of Guizhou University (No. EAE-GZU-2021-E025, Guiyang, China; 30 March 2021). Effective procedures were implemented to reduce pain and distress, and overall health, zoonotic infections, and pathogenic microbial infectious diseases were all thoroughly controlled and monitored.

### Experimental animals

Four healthy Qianbei Ma billy goats were randomly selected at 3 months of age (average weight 17.21 kg) and 9 months of age (average weight 32.71 kg) at Fuxing Herd Co., in Zunyi City, Guizhou Province, China. Through intravenous injection with propofol (a dose of 0.5 mL/kg), the goats were stunned. Then, the veterinarian quickly dissected the scrotum with scissors and forceps to obtain a testicular tissue sample. The semen parameters collected before the testicles were taken showed that the 9-month-old goat group displayed normal spermatogenesis. One of the testes was randomly selected for RNA isolation and histological analysis. Testicular samples were immediately cut into small pieces, transferred to cryogenic vials, and stored in liquid nitrogen. The samples were shipped to LC Sciences in Hangzhou, China, for subsequent library construction and sequencing.

### Morphology and tissue evaluation

Sections of testicular tissue were made. The sections were routinely deparaffinized with xylene, dewaxed in water for 3 min each with 100% ethanol, 95% ethanol, 80% ethanol and 70% ethanol, stained with hematoxylin for 5 min, washed with water, stained with 1% hydrochloric acid in ethanol for 5 s, rinsed with tap water for 20 min, stained with eosin for 30 s, washed with water, dehydrated for 3 min each with 95% and 100% ethanol, sealed with neutral gum after drying.CaseViewer2.4 software was used to scan the image and save it. Image-Pro Plus 6.0 analysis software was used to measure the area and diameter of the intact convoluted seminiferous tubules in the sections (standard unit: millimeters). At last, counting different types of cells to calculate cell number per unit area.

### ELISA detection

Blood was collected from the goats by the neck blood collection method and centrifuged at 3000 r/min for 10 min at 4 °C, and the serum was separated. The levels of testosterone (T), luteinizing hormone (LH), follicle stimulating hormone (FSH) and anti-Müllerian hormone (AMH) were determined using the goat ELISA kit provided by Zhiqin Tiancheng Biological Company.

### Total RNA extraction, purification and sequencing

High-quality RNA was isolated from testicular samples using the TRIzol method according to the manufacturer’s (Invitrogen) instructions. The concentration of RNA was determined by a NanoDrop spectrophotometer, and the integrity of the RNA was determined with an Agilent 2100 Bioanalyzer. The PCR products were purified (AMPure XP system), and the library quality was assessed on an Agilent Bioanalyzer 2100 system. The Illumina NovaSeq platform was selected to sequence the library. Raw reads were filtered, and Q30 and GC content were calculated to obtain clean reads for subsequent analysis. Differential expression analysis of the two groups was performed using DESeq2, and | log2 (FoldChange)| >1 and p value < 0.05 were set as thresholds for significantly different expression.

### GO and KEGG enrichment analysis

Gene Ontology (GO) enrichment analysis of DEGs and statistical enrichment analysis of DEGs in Kyoto Encyclopedia of Genes and Genomes (KEGG) pathways were performed using the online analysis tool (http://www.bioinformatics.com.cn/). GO terms and KEGG pathways were considered significantly enriched when *P* < 0.05.

### Quantitative real-time PCR

To verify the reliability of the sequencing analysis results, the relative abundance of mRNAs of the six selected transcripts was determined by qRT‒PCR. Based on the sequences in the NCBI database, the corresponding primers were designed using Primer Premier 5.0 (Premier Inc., Canada), and the primer sequences and parameters are shown in Table 1. Primer synthesis was performed by Shanghai Sangong Bioengineering Co. cDNA was used as the amplification template for qRT‒PCR based on the SYBR Green method. The qRT‒PCR amplification system (10 µL) contained 0.5 µL of upstream and downstream primers, 0.5 µL of template, 5 µL of 2X SYBR mastermix, and 3.5 µL of enzyme-free water. Three replicates were set up for each sample, and a blank control of 3 replicates was set up at the same time. qRT‒PCR was carried out in accordance with the following conditions: 95 °C for 2 min; 95 °C for 15 s, the corresponding annealing temperature (Table 1) for 15 s, 72 °C for 1 min, and fluorescence signal acquisition after the completion of extension for 40 cycles. Fluorescence signal acquisition was performed after completion of extension. β-Actin was used as an internal reference.

**Table 1:** Detailed information of each primer

Gene Name	NCBI ID	Primer sequence	Products size (bp)	Annealing Temperature (℃)
*DKK1*	XM_005698161.3	F:AGCGTTGTTACTGTGGAGAAGGTC	73	61.6
		R:CTGGAAGAATTGCTGGCTTGATGG		
*WFDC8*	XM_018056874.1	F:ACAAGAGCCCCACTAATG	146	59.7
		R:GATTTTGTGGTCTGTATTGC		
*PRSS58*	XM_005701261.2	F:TTATGAATTTAATCCTTCTGTG	126	59.7
		R:CACAAGGCAAGTAATCAGAC		
*ECM1*	XM_013962438.2	F:TTGGTCTTGGCCTGTTTGAC	121	59.7
		R:CAGCATAGCCCACTTCTTGA		
*LHCGR*	NM_001314279.1	F:TCACACTGGAAAGATGGCACACC	128	59
		R:AAGAGGCAGCATGGCGATGAG		
*CRB2*	XM_018055744.1	F:TGGACGAATGCCTGTCAGAG	170	59
		R:GAAGGTAGGGATGCAGGTGG		
*β-actin*	XM_018039831.1	F:TGATATTGCTGCGCTCGTGGT	266	60
		R:GTCAGGATGCCTCTCTTGCTC		

### Statistical analysis

All data are presented as means ± SD of three biological replicates and three technical replicates to ensure the accuracy of the experimental data. Data was processed by SPSS(V25.0) software. The difference between the two groups was determined by t test, and *P* < 0.05 was considered statistically significant.

## Results

### Histomorphometric analysis of the Qianbei Ma goat testes before and after sexual maturity

**Fig. 1:**
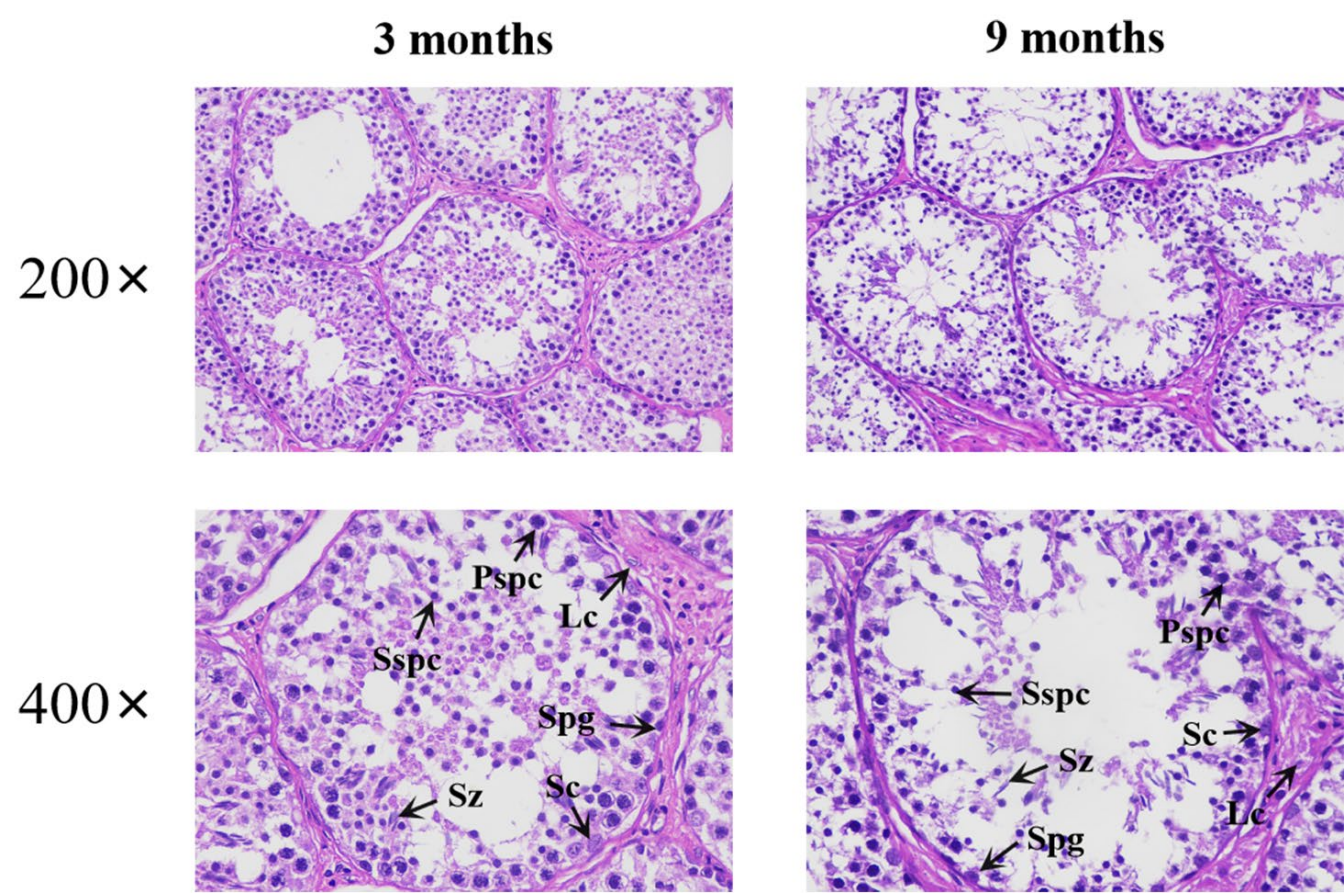
HE staining of testicular tissues at different months of age. Note: LC:Leydig cell; Sc:Sertoli cell; Sz:spermatozoon; Spg:spermatogonia; Pspc:primary spermatocyte; Sspc:secondary spermatocyte

The testicular tissue of the Qianbei Ma goat consisted of spermatogenic tubules and testicular interstitial parts (Fig. 1). In terms of the size of the spermatogenic tubules, the area and diameter of the tubules were larger at 9 months than at 3 months. There was an increase in the number of spermatocytes, interstitial cells, spermatogonia, and secondary spermatocytes in the lumen of the tubules compared to at 3 months of age. Spermatozoa were observed in the lumen of the spermatogonial tubules at 3 months of age, indicating that puberty had begun in the Qianbei goats. The number of interstitial cells in the testicular tissue increased at 9 months of age compared to 3 months, while the number of Sertoli cell in the testis decreased at 9 months of age compared to 3 months. There was also an increase in secondary spermatogonia, but the difference was not significant (Table 2).

**Table 2:** Histomorphologic physicochemical indices of testis

Items	Different months	
	3	9
Seminiferous tubule area (mm)	0.052 ± 0.011	0.068 ± 0.02
Diameters of seminiferous tubules (mm^2^)	0.254 ± 0.023	0.289 ± 0.038
Sertoli cell	14 ± 5.292	5.667 ± 0.577
Leydig cell	38.667 ± 7.024	51.667 ± 19.757
spermatozoon	27.667 ± 11.15	42.333 ± 20.232
spermatogonia	17.667 ± 11.676	27.667 ± 6.658
primary spermatocyte	27.667 ± 22.898	9 ± 2.646
Secondary spermatocyte	25.667 ± 7.767	28.333 ± 15.631

### Expression levels of reproductive hormones before and after sexual maturity

**Fig. 2:**
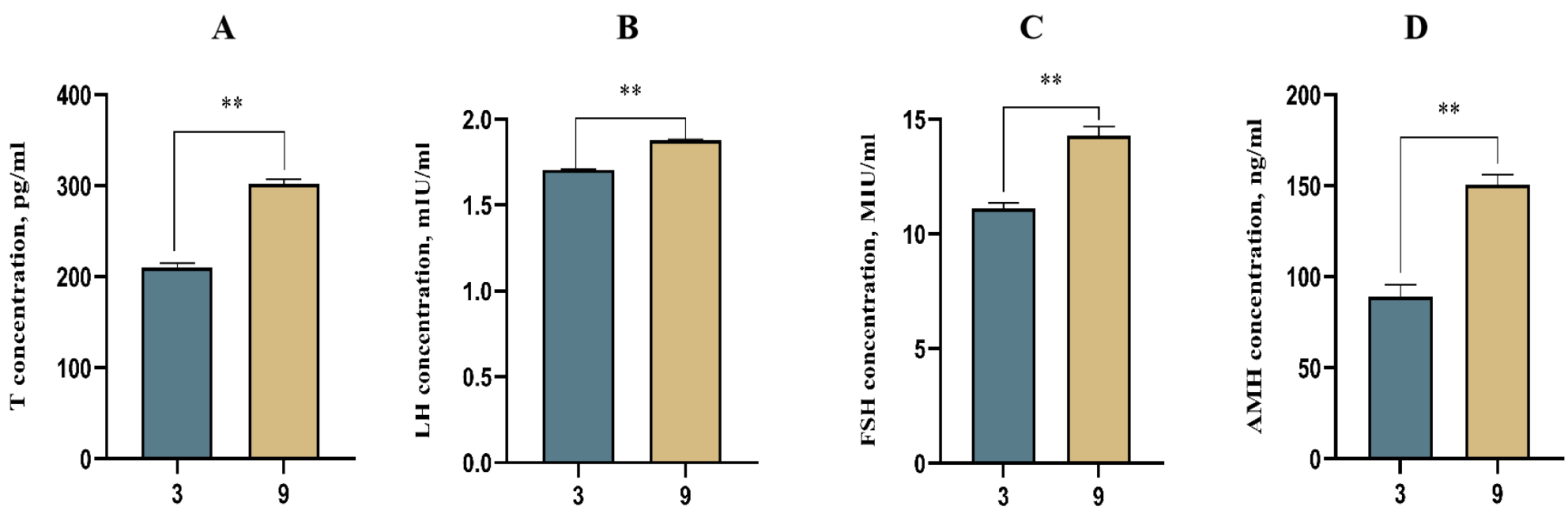
Hormone measurements at different months of age. (*n* = 4). Note: (**A**):Testosterone(T) level; (**B**):luteinizing hormone(LH) level; (**C**):Follicle stimulating hormone(FSH) level;D:Anti Mueller hormone(AMH) level. ** indicate highly significant differences(*P*<0.01);* indicate significant differences(*P*<0.05)

The ELISA results showed (Fig. 2) that the content of each hormone level at 9 months of age was significantly higher than that at 3 months of age (*P* < 0.01). The testosterone hormone content was 209.85 ng/ml at 3 months of age and 301.90 ng/ml at 9 months of age (Fig. 2A); luteinizing hormone was secreted at 1.71 mIU/ml at 3 months of age and 1.88 mIU/ml at 9 months of age (Fig. 2B); and follicle stimulating hormone was secreted at 11.10 mIU/ml at 3 months of age and 14.28 mIU/ml (Fig. 2C). The anti-Müllerian hormone content was 89.30 ng/ml at 3 months of age and 150.70 ng/ml at 9 months of age (Fig. 2D).

### Total RNA quality testing and data quality control analysis

Eight samples were selected from the 3-month-old control group (M1, M2, M3, M4) and the 9-month-old experimental group (S1, S2, S3, S4). By sequencing, an average of 44,687,384 bases was obtained. After quality control, an average of 43,202,260 clean reads were generated, which for the experimental group was between 4,198,8148 and 4,631,0130 and for the control group was between 39,167,034 and 47,103,696. The average Q30 values for the experimental and control groups were 93.25% and 93.24%, respectively, satisfying the 90% requirement for Q30. The average GC content mean values of the experimental and control groups were 51.85% and 52.14%, respectively, indicating that the quality of the sequencing results was good enough to support all analyses of the expression profiles obtained from the sequencing data (Table 3).

**Table 3:** Quality control and sequencing data statistics

Sample	Raw_Reads	Raw_Bases	Clean_Reads	Clean_Bases	Q30	GC
M1	47,800,424	7.17G	46,310,130	6.95G	93.15	52.23
M2	43,286,288	6.49G	41,988,148	6.3G	93.23	51.82
M3	45,706,346	6.86G	44,102,186	6.62G	93.33	51.76
M4	45,216,384	6.78G	43,909,240	6.59G	93.16	51.64
S1	43,452,350	6.52G	42,052,984	6.31G	93.08	52.06
S2	43,716,118	6.56G	42,185,962	6.33G	93.37	51.92
S3	45,338,010	6.8G	43,697,500	6.55G	93.47	51.61
S4	46,174,996	6.93G	44,884,950	6.73G	93.28	51.72

### Differential expression analysis of Qianbei Ma goat testes before and after sexual maturity

**Fig. 3:**
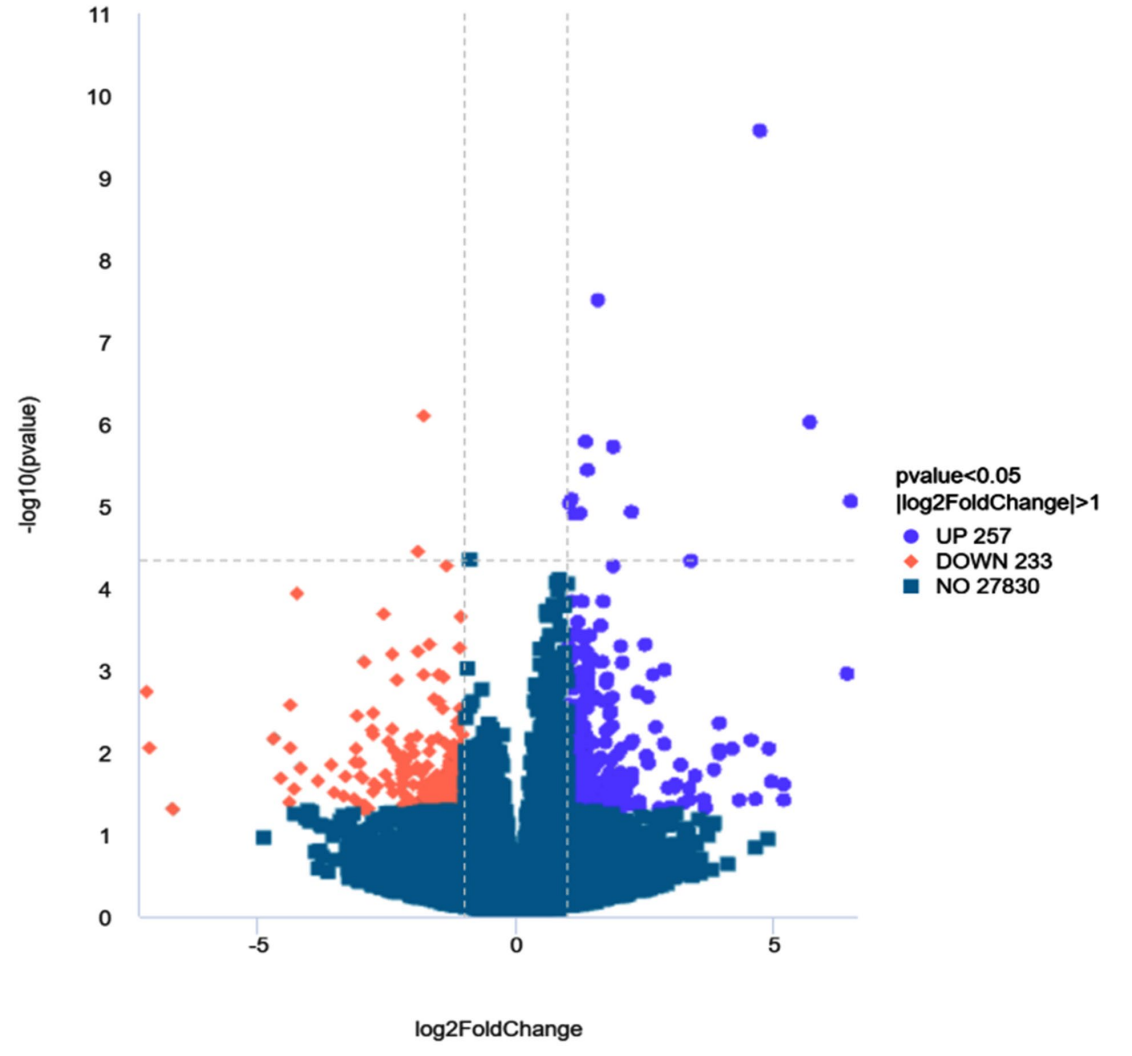
Differential mRNA expression between experimental and control groups. Purple dots indicate genes that were significantly 257 up-regulated, orange dots indicate genes that were significantly 233 down-regulated, while blue dots indicate genes with no difference

To explore the role of mRNA in the regulation of testicular development in Qianbei Ma goats, we assessed a total of 27,830 genes, and the detected genes were differentially expressed using |log2Foldchange| > 1 and P value < 0.05 as the criteria. A total of 490 genes were differentially expressed, of which 233 genes were upregulated and 257 genes were downregulated (Fig. 3).

### GO and KEGG enrichment analysis

**Fig. 4:**
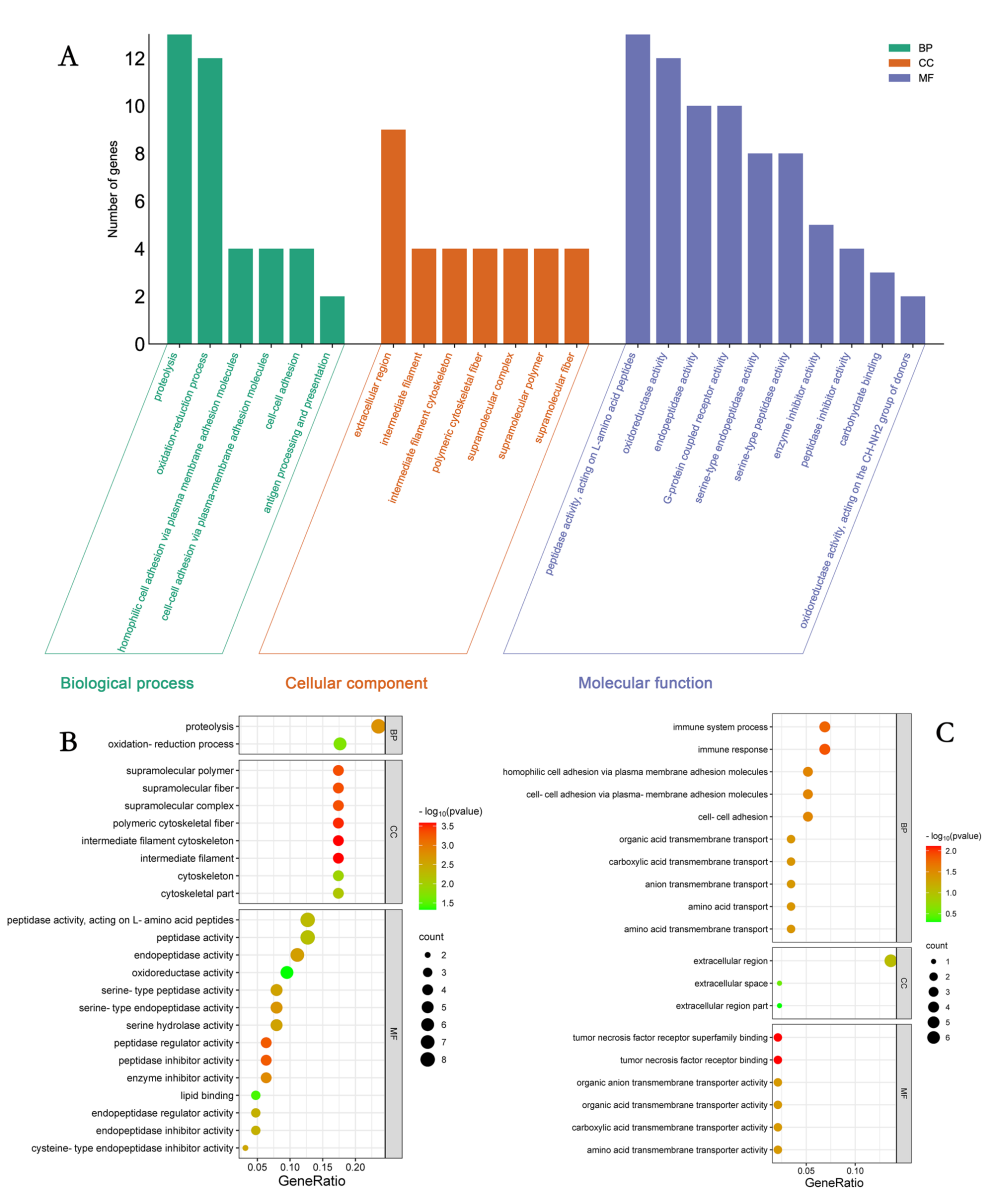
DEGs GO enrichment analysis of 3-month-old and 9-month-old Qianbei sheep, (**A**): ALL DEGs GO enrichment analysis graph, horizontal coordinate is GO items, vertical coordinate is genes enriched by GO items; (**B**): UP DEGs GO enrichment bubble graph, (**C**): DOWN DEGs GO enrichment bubble graph

The 490 differentially expressed DEGs screened were analyzed by GO functional clustering and classified into biological processes (BPs), cellular components (CCs) and molecular functions (MFs), with a total of 23 semantics enriched. Among them, biological process components accounted for 26.53%, cellular components accounted for 22.45%, and molecular functions accounted for 51.02%. The differentially expressed genes were mainly enriched in molecular functional biological processes (Fig. 4A). The upregulated differentially expressed genes were mainly involved in molecular functions, of which the prominent enriched terms were the peptidase activity pathway and transferase activity pathway (Fig. 4B). The downregulated differentially expressed genes participated in biological processes, and the prominent enriched terms were extracellular region functions (Fig. 4C).

**Fig. 5:**
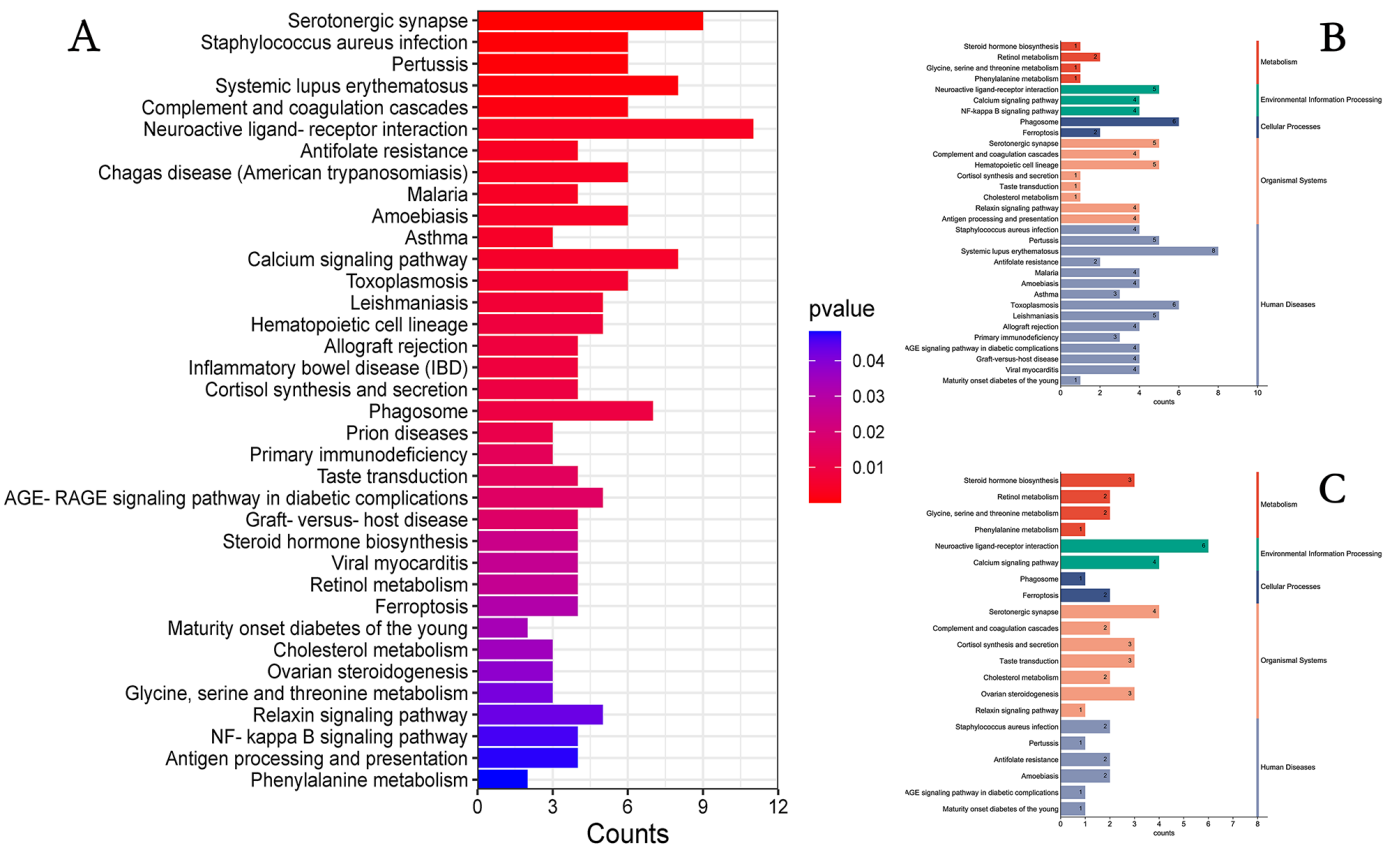
KEGG enrichment analysis of 3-month-old and 9-month-old DEGs in Qianbei hemp sheep; (**A**) ALL DEGs KEGG enrichment analysis plot, horizontal coordinates are KEGG pathway terms, vertical coordinates are genes enriched by KEGG terms; (**B**): UP DEGs KEGG enrichment plot, (**C**): DOWN DEGs KEGG enrichment plot

**Fig. 6:**
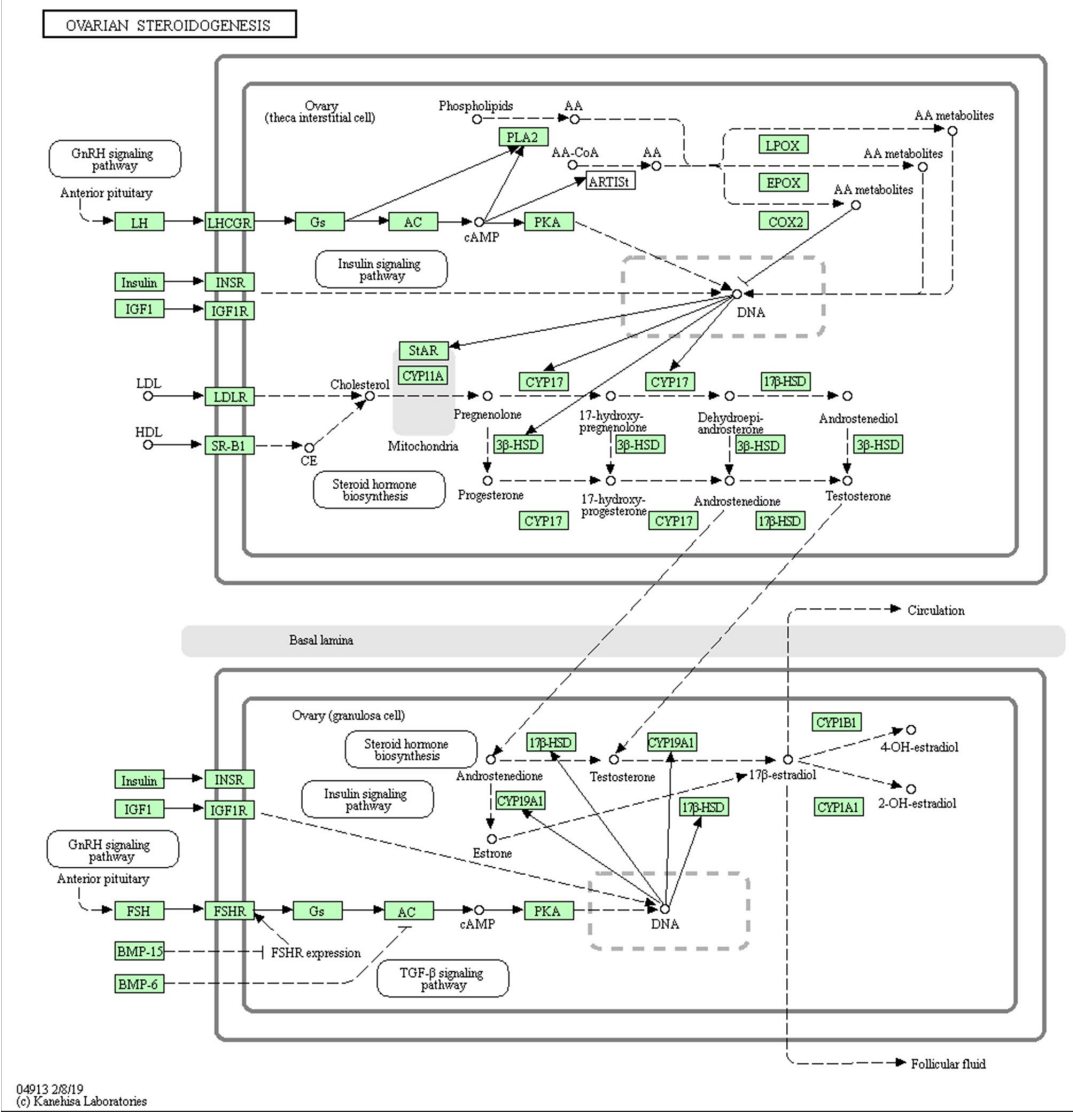
The KEGG steroid hormone biosynthesis

The KEGG analysis showed that 194 pathways were enriched by 490 DEGs in both groups. When a P value < 0.05 was chosen as the threshold for significant enrichment, we identified the 36 most significantly enriched pathways (Fig. 5A); these pathways included serotonergic synapses, steroid hormone biosynthesis, neuroactive ligand‒receptor interactions, relaxin signaling pathway, NF-KB signaling pathway, and others. By performing KEGG enrichment analysis on the upregulated DEGs, we identified 23 pathways (*P* < 0.05), of which the main enrichment was in the disease pathway section (Fig. 5B). Performing KEGG enrichment on downregulated DEGs, we identified 36 pathways (*P* < 0.05) that were predominantly enriched in the organic systems section (Fig. 5C). To screen for the effects of testicular development and androgen secretion in goats, combining GO and KEGG analyses, we identified several pathways associated with cell growth and hormone secretion. These were neuroactive ligand‒receptor interaction, calcium signaling pathway, steroid hormone biosynthesis,and Wnt signaling pathway. and the DEGs *LHGCR* is highly enriched in steroid hormone biosynthesis(Fig. 6).In addition, we also obtained upregulated DEGs *CRB2*, *WFDC8*, *PRSS58* and downregulated DEGs *DKK1* and *ECM1*.

### RNA-seq data validation

**Fig. 7:**
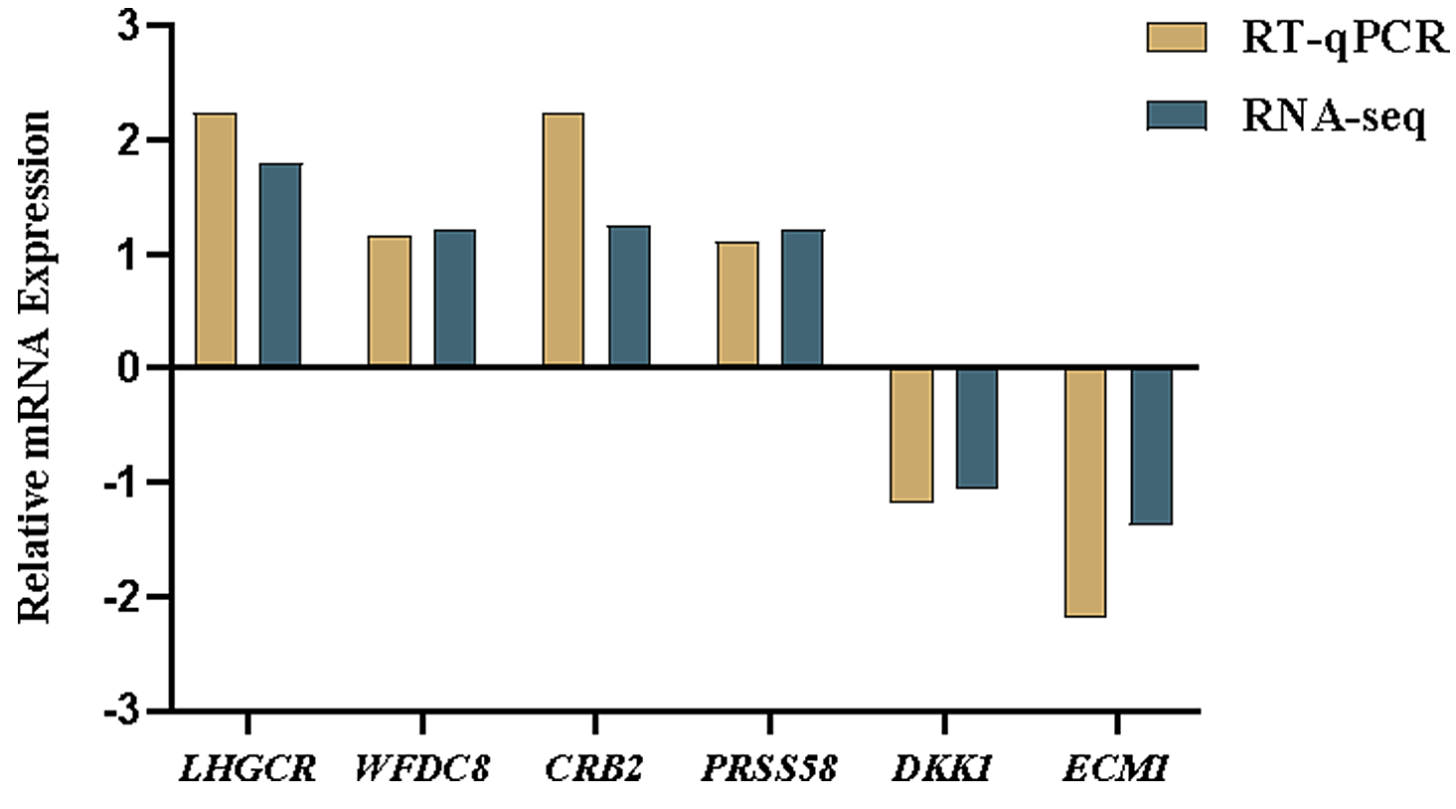
Validation of DEGs by RT-qPCR

To validate the RNA-Seq results, the relative abundance of four upregulated genes, *LHCGR*, *CRB2*, *PRSS58*, and *WFDC8*, and two downregulated genes, *DKK1* and *ECM1*, were examined by RT‒qPCR, and the RT‒qPCR expression patterns of the selected genes were consistent with the results of the RNA-Seq analyses (Fig. 7). The PCR products were subjected to Sanger sequencing, verifying that the selected genes had the correct cyclization linker site. These results indicated that the genes sequencing data were reliable, and could be used for further analysis.

## Discussion

Goats are economically important animals, and the size and development of testes in males directly determine fertility, influencing the economic benefits of the goat industry [17, 18]. In general, there is a complex regulatory relationship between genotype and biological phenotype [19]. mRNA is one of the important forms of RNA in living organisms, which is formed by the transcription of coding genes followed by shear modification and is subsequently transported to the cytoplasm to be translated into functional proteins to perform biological functions [20]. Therefore, understanding the regulation of genes is crucial in the growth and development of animals. In recent years, RNA-seq technology and bioinformatics have been utilized to analyze the expression levels of all gene transcripts at different time points. These genes are up- or downregulated at different levels of proteins and metabolites, which can induce phenotypic changes in animals. For example, 302 DEGs were screened for seasonal reproduction in male lionhead geese, and the *HOX* gene was determined to be important for the regulation of testicular development between the nonbreeding and breeding periods in lionhead geese [21]. A total of 5068 DEGs were screened for testicular sexual maturation in large white pigs and Tongcheng pigs, and candidate genes such as *TRIP13*, *NR6A1*, *STRA8*, *PCSK4*, *ACRBP*, *TSSK1*, and *TSSK6* were selected regarding testicular maturation [22].

The testicular seminiferous tubules are important sites for spermatogenesis and contain cells such as spermatogonia, spermatocytes, spermatids, and supporting cells [23]. Our analysis of testicular tissue sections before and after sexual maturity in Qianbei Ma goats revealed that the area and diameter of the seminiferous tubules increased with age, and the number of supporting cells changed only slightly. Some researchers have found that testicular supporting cells usually mature before the primiparous stage and lose their proliferative ability after maturation, after which they are maintained at a dynamic level [24]. We observed that spermatozoa had already appeared in 3-month-old slices, while other studies have shown that sexual maturity can be reached at 4 months of age in Qianbei Ma goats [25], indicating that puberty has already begun in the Qianbei Ma goats. This result is consistent with that of Ren et al. [26], who observed changes in testicular tissue morphology in 3-month-old dairy goats.

Testosterone (T) is an important steroid hormone that regulates growth and development and sperm synthesis in males [27]. Luteinizing hormone (LH) promotes testicular development and eventual maturation of spermatogenesis [28], and follicle-stimulating hormone (FSH) has a major role in males by acting synergistically with testosterone to maximize sperm production [29]. Studies have shown that AMH levels are positively correlated with total sperm count, concentration and positive motility [30]. In this study, the serum levels of these hormones were determined at two different months of age in Qianbei Ma goats, and the levels at age 9 months were all extremely significantly higher than those at age 3 months (*P* < 0.01), which was similar to the study of Zhang [7]. This indicates that before puberty, the reproductive organ is not well developed and cannot secrete more reproductive hormones. After sexual maturity, the reproductive organs of Qianbei Ma goats gradually developed completely, and most of the reproductive cells were able to secrete more reproductive hormones, thus gaining fertility.

We further investigated the molecular mechanism of testicular development before and after sexual maturity in Qianbei Ma goats using RNA-seq and identified a total of 27,830 genes. A total of 490 DEGs were detected in the comparison of the 2 groups, of which 233 were upregulated and 257 were downregulated. To explore the role of DEGs in the development of testes before and after sexual maturity in Qianbei Ma goats, we performed functional annotation by GO, which revealed that the differentially expressed genes were enriched in peptidase activity. Peptidase activity is an important physiological activity in the development of organisms [31]. It has been shown that acid peptidase activity released from in vitro cultured porcine embryos is positively correlated with late development and embryo quality [32]. Serine proteases (*PRSS*), which have a nucleophilic Ser residue at the active site, constitute nearly one-third of all known proteases, many of which have been identified as testis-specific proteases and play an important role in sperm development and male reproduction [33–35].*PRSS58*, is a gene in a family of serine proteases,May be involved in the biological functions of the testes. *CRB2* is a member of a family of cysteine- and glycine-enriched proteins that mediate protein‒protein interactions with significant roles in cell growth, expansion, and differentiation [36]. *WFDC8* is whey acidic protein tetrasulfide bond core protein 8, which is one of the proteins that regulates sperm maturation and contributes significantly to the immune function of the male reproductive tract [37, 38]. The upregulated differential gene PRSS58, which was significantly enriched in enzyme peptide activity by GO functional analysis in this study, may also be a potential candidate gene in sperm development. The GO-enriched downregulated DEGs were *ECM1* and *DKK1*. *ECM1* is a glycoprotein and a sperm detection marker product [39]. *DKK1* is a WNT signaling antagonist, and the WNT signaling pathway contributes to sperm development through the promotion of ovarian development and inhibition of testicular development in the early gonad, which plays an important regulatory role in mammals [40]. Overall, GO term-enriched DEGs may be involved in regulating the biological processes of testicular development and spermatogenesis in Qianbei Ma goats. *LHCGR* is a specific receptor for luteinizing hormone [41], which acts to mediate the synthesis and secretion of androgens and growth factors by testicular mesenchymal stromal cells in male animals [42]. Sun et al. (2011)correlated polymorphisms of GnRH and *LHCGR* genes with semen quality in cattle and found that the sperm density of individuals with the TT genotype at the G651656T locus of the *LHCGR* gene was significantly higher than that of the GT genotype [43]. Liu et al. (2009) analyzed the polymorphisms of the 5′UTR and three exons of the LHβ gene in Simmental and Charolais cows by PCR-SSCP and correlated them with semen quality traits [44]. They found that there was an SNP in exon 2, and the mutation was significantly correlated with the deformity rate of frozen semen and ejaculation volume, which proved that the *LHCGR* gene could be a candidate gene influencing semen quality. KEGG enrichment results showed that *LHCGR* was significantly enriched in pathways related to testicular development and androgen secretion processes, such as neuroactive ligand‒receptor interaction, calcium signaling pathway, cortisol synthesis and secretion, cAMP pathway, and ovarian steroid synthesis and secretion. For example, the classical cAMP signaling pathway has an important role in promoting testosterone synthesis and cell proliferation and regulating cellular homeostasis [45–47]. Therefore, the KEGG pathway enriched by reproduction-related genes plays an important role in testicular development and spermatogenesis in Qianbei Ma goats.

## Conclusion

The dynamic changes in testicular development and spermatogenesis between pre- and postsexual maturity in Qianbei Ma goats are characterized by the sex ligand‒receptor interaction pathway, calcium signaling pathway and response to steroid hormone biological processes. *PRSS58*, *ECM1*, *WFDC8* and DEGs of *LHCGR* may be the key genes regulating the molecular mechanisms of sexual maturation in Qianbei Ma goats. However, their specific regulatory mechanisms still need to be further studied.

## Data Availability

The datasets presented in this study can be found in online repositories. Thename of the repository/repositories and accession numbers can be found atSequence Read Archive (SRA) repository, PRJNA1014973.

## Abbreviations

DEGs: Differentially expressed genes
LH: luteinizing hormone
FSH: follicle stimulating hormone
T: testosterone
AMH: anti-Müllerian hormone
GO: Gene Ontology
KEGG: Kyoto encyclopedia of genes and genomes
qRT‒PCR: Quantitative real-time PCR
BPs: Gene ontology biological processes
CCs: Gene ontology cellular components
MFs: Gene ontology molecular functions
